# Management of Unruptured Infectious Intracranial Aneurysms in Infective Endocarditis: A Case Report and Literature Review

**DOI:** 10.7759/cureus.76636

**Published:** 2024-12-30

**Authors:** Anh Tran Hue, Tatsuya Tanaka, Fumitaka Yamane, Hiroshi Itokawa, Kimihiro Nakahara, Akira Matsuno

**Affiliations:** 1 Department of Neurosurgery, International University of Health and Welfare Narita Hospital, Narita, JPN; 2 Department of Neurosurgery, International University of Health and Welfare Atami Hospital, Atami, JPN

**Keywords:** acute ischemic stroke, cerebral infarction, coil embolization, endovascular therapy, infective endocarditis, intracranial infectious aneurysm, valvular disease

## Abstract

Infectious intracranial aneurysms (IIAs) are rare lesions with fragile arterial walls located within the aneurysms, carrying a high risk of rupture. Standard management often involves antibiotic therapy and parent artery occlusion; however, the latter carries a significant risk of cerebral infarction. This report presents a case of an unruptured IIA following cerebral infarction, successfully treated with coil embolization while preserving the parent artery.

A 54-year-old man with a history of smoking and alcohol consumption presented with fever and impaired consciousness. Upon admission, he exhibited signs of sepsis and meningitis, with a Glasgow Coma Scale score of 8. Imaging revealed multiple cerebral infarctions alongside embolic lesions in the kidney and spleen. Transthoracic echocardiography confirmed mitral valve infective endocarditis. The patient was started on antibiotics and underwent mitral valve repair on hospital day 4. On hospital day 44, MRI and magnetic resonance angiography (MRA) identified a 5-mm unruptured aneurysm in the left middle cerebral artery, consistent with an IIA. Coil embolization was performed on hospital day 60 under general anesthesia, achieving complete aneurysm obliteration without compromising the parent artery. Postoperatively, the patient experienced no new infarctions and demonstrated a favorable recovery, leading to discharge on hospital day 110. Follow-up MRI and MRA performed 18 months post-treatment confirmed the absence of aneurysm recurrence.

This case highlights the importance of individualized treatment strategies in managing IIAs. While parent artery occlusion remains the standard approach, coil embolization offers a viable alternative in select cases, particularly for preserving parent artery integrity and reducing the risk of cerebral infarction.

## Introduction

Infectious intracranial aneurysms (IIAs) are rare but potentially life-threatening complications of infective endocarditis (IE), occurring in approximately 2-10% of cases [1-4]. Timely diagnosis and management are crucial, as the rupture of an IIA can lead to catastrophic outcomes [5-7]. Systemic antibiotic therapy is the primary treatment for unruptured IIAs and can often result in aneurysmal shrinkage or resolution [5-7]. However, conservative treatment alone is associated with high mortality in cases of rupture or progressive aneurysmal enlargement, necessitating endovascular or surgical intervention [5-7].

IIAs are commonly located in peripheral cerebral arteries and are characterized by fragile vessel walls, a fusiform shape, and small size [8]. These features often require parent artery occlusion during endovascular treatment [8], which carries a significant risk of ischemic complications. To mitigate this risk, various treatment strategies have been applied, including coil embolization, aneurysm resection with bypass surgery, and pre-occlusion balloon test occlusion [9-11]. Despite these advances, an optimal treatment strategy for IIAs remains undefined, highlighting the need for individualized approaches.

This report describes a case of an unruptured IIA that developed following a cerebral embolism secondary to IE. The case highlights the success of coil embolization in preserving parent artery integrity and includes a review of the literature on unruptured IIAs, focusing on treatment indications and management strategies.

## Case presentation

A 54-year-old male presented to the emergency department with a fever and impaired consciousness that had developed over several days. The patient’s medical history includes disc herniation, hepatitis C, a 10-cigarette-per-day smoking habit, and regular alcohol consumption of approximately 120 g four times per week. Upon admission, body temperature was 39.2°C, heart rate 120 beats per minute, blood pressure 130/91 mmHg, respiratory rate 24 breaths per minute, and percutaneous oxygen saturation 99% on room air. Neurological examination revealed a Glasgow Coma Scale (GCS) score of 8 (E3V2M3), neck stiffness, and no limb paralysis. The oral examination noted multiple dental caries and tooth loss.

Laboratory investigations revealed leukopenia (WBC count, 3,450/μL), thrombocytopenia (platelet count, 38,000/μL), elevated inflammatory markers (CRP, 27.4 mg/dL), and coagulopathy (D-dimer, 30.8 μg/mL). CSF analysis showed a cell count of 154/μL, protein level of 122.2 mg/dL, and glucose level of 39 mg/dL. The initial laboratory tests are listed in Table 1.

**Table 1:** Laboratory test on initial presentation. MCV: Mean Corpuscular Volume; MCHC: Mean Corpuscular Hemoglobin Concentration; RDW: Red Cell Distribution Width; MPV: Mean Platelet Volume; BUN: Blood Urea Nitrogen; ALT: Alanine Aminotransferase; AST: Aspartate Aminotransferase.

Laboratory test	Result	Normal range
WBCs	3,450 /μL	4,000-8,000 /μL
RBCs	4.30 x 106/μL	4.35-5.55 x 106/μL
Hemoglobin	13.6 g/dL	13.7-16.8 g/dl
Hematocrit	38.10%	40.7-550.1 %
MCV	88.6 fL	83.6-98.2 fl
MCHC	35.70%	31.7-35.3 %
RDW	14.0 fL	11.8-14.5 fL
MPV	11.5 fL	8-12 fL
Platelet Count	38,000 /μL	140,000-340,000/μL
Neutrophils	93%	38.5-80.5%
Lymphocytes	3.00%	16.5-49.5 %
Monocytes	3.30%	2.0-10.0%
Eosinophils	0.00%	0.0-8.5 %
Basophils	0.60%	0.0-2.5 %
Glucose	113 mg/dL	73-109 mg/dL
BUN	14.9 mg/dL	8.0-20.0 mg/dL
Creatinine	1.17 mg/dL	0.65-1.07 mg/dL
Sodium	136 mEq/L	138-145 mEq/L
Potassium	3.7 mEq/L	3.6-4.8 mEq/L
Chloride	103 mEq/L	101-108 mEq/L
Calcium	8.1 mg/dL	8.8-10.1 mg/dL
Phosphorous	1.8 mg/dL	2.7-4.6 mg/dL
Protein, Total	5.4 g/dL	6.6-8.1 g/dL
Albumin	2.5 g/dL	3.4-5.4 g/dL
Bilirubin, Total	3.3 mg/dL	0.4-1.5 mg/dL
ALT	58 U/L	10-42 U/L
AST	101 U/L	13-30 U/L
Alkaline Phosphatase	72 U/L	38-113 U/L
Lactate	0.75 mmol/L	0.7-2.1 mmol/L
C-Reactive Protein	27.40 mg/L	0.00-0.14 mg/dL
Procalcitonin	9.43 ng/mL	<0.05 ng/mL
D-dimer	30.8 μg/mL	<1.0 μg/mL
CSF, cell count	154 /μL	－
CSF, protein	122.2 mg/dL	10.0-35.0 mg/dL
CSF, glucose	39 mg/dL	50-80 mg/dL

**Figure 1:**
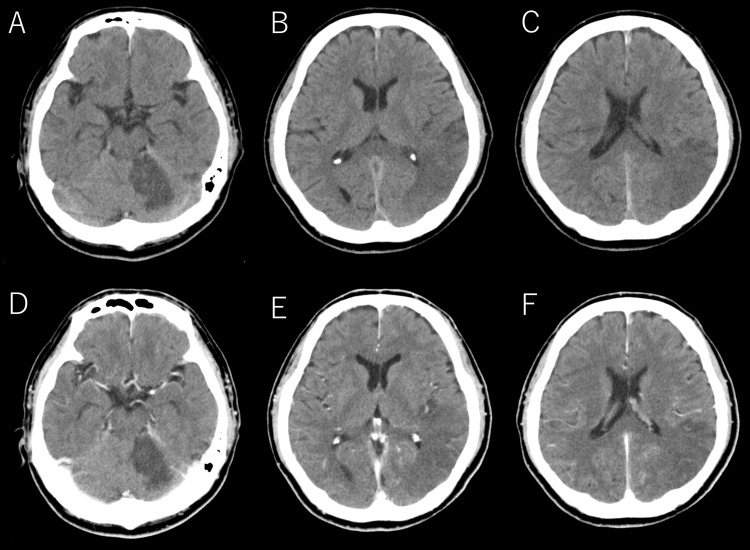
Initial head CT. (A, B, C): Non-contrast head CT images; (D, E, F): Contrast-enhanced head CT images. Multiple infarcts are observed in the left cerebellum, left temporal lobe, and parietal lobe. No evidence of brain tumors, brain abscesses, venous sinus thrombosis, or major cerebral artery occlusion is detected.

A CT scan of the head, performed without the intravenous administration of contrast material, showed hypodense areas in the left cerebellar hemisphere and left parietal-occipital region, consistent with cerebral infarction (Figure 1).

**Figure 2:**
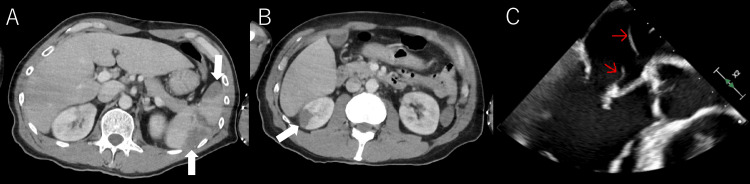
Abdominal CT and transthoracic echocardiography findings. (A, B): Contrast-enhanced abdominal CT reveals infarctions in the spleen and right kidney (white arrows). (C): Transthoracic echocardiography shows two threadlike to bandlike vegetations exceeding 30 mm attached to the anterior mitral leaflet (red arrows).

Contrast-enhanced thoracoabdominal CT showed wedge-shaped hypodense areas in the right kidney and spleen, consistent with infarctions (Figures 2A-2B). Transthoracic echocardiography revealed two 30-mm linear vegetations on the anterior leaflet of the mitral valve, confirming mitral valve infective endocarditis (Figure 2C).

Blood culture was positive for Methicillin-sensitive *Staphylococcus aureus* (MSSA).

The patient was diagnosed with sepsis, infective endocarditis, systemic embolism, and meningitis, likely originating from dental caries. Initial treatment included vancomycin, ceftriaxone, ampicillin, acyclovir, and dexamethasone. On day 4, mitral valve repair was performed to prevent further embolization, and MSSA was again isolated from the vegetation. Antibiotic therapy was subsequently adjusted to cefazolin on day 8, later changed to meropenem on day 47, and oral cephalexin on day 102. Anticoagulation therapy with aspirin and warfarin was initiated to reduce the risk of further embolization.

**Figure 3:**
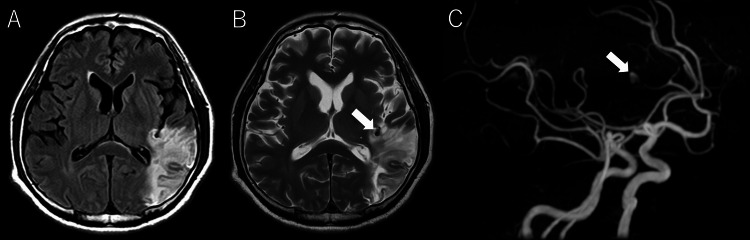
Post-mitral valve repair head MRI/MRA. (A) FLAIR image; (B) T2-weighted image; and (C) MRA. Chronic cerebral infarcts are observed, along with an aneurysm in the left middle cerebral artery (arrow). MRA: Magnetic Resonance Angiography; FLAIR: Fluid-Attenuated Inversion Recovery image.

The patient’s level of consciousness improved to a GCS score of 14. However, due to the presence of aphasia and right hemispatial neglect, the patient was kept hospitalized for rehabilitation purposes. On day 44, magnetic resonance angiography (MRA) detected a newly developed unruptured cerebral aneurysm in the left middle cerebral artery (MCA) (Figure 3).

**Figure 4:**
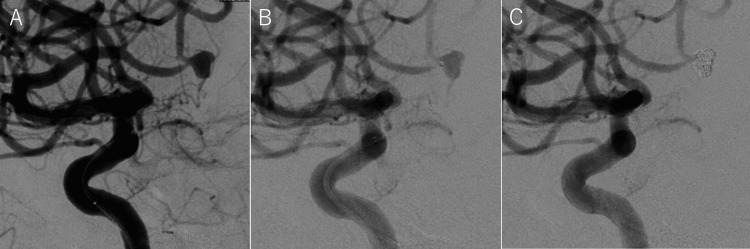
Coil embolization for the cerebral aneurysm. (A) Digital subtraction angiography (DSA) reveals an aneurysm with a maximum diameter of 5.4 mm in the M2-3 segment of the left middle cerebral artery. (B) Microcatheter placement within the aneurysm. (C) Post-coil embolization, no visualization of the aneurysm is evident.

Digital subtraction angiography (DSA) characterized the aneurysm as having a neck measuring 1.5 mm, a long axis of 5.4 mm, and a short axis of 3.8 mm, consistent with an infectious aneurysm (Figure 4A).

To prevent aneurysm rupture, endovascular coil embolization was performed on day 60. Under general anesthesia, an 8-Fr balloon guiding catheter (Optimo; Tokai Medical Products, Aichi, Japan) was positioned in the left internal carotid artery. Using a distal access catheter (Tactics; Technocrat Corporation, Aichi, Japan), a microcatheter (Headway DUO; Terumo Medical Corporation, Tokyo, Japan), and a microguidewire (Synchro SELECT Soft; Stryker, Fremont, CA, USA), the aneurysm was accessed (Figure 4B). Coiling was initiated with a Target 360 Ultra (3 mm × 10 cm; Stryker) to establish a frame, followed by three additional coils (Hyper Soft 3D and i-ED COIL Complex Infini; Terumo and Kaneka Medix, Osaka, Japan), achieving complete aneurysm occlusion (Figure 4C).

**Figure 5:**
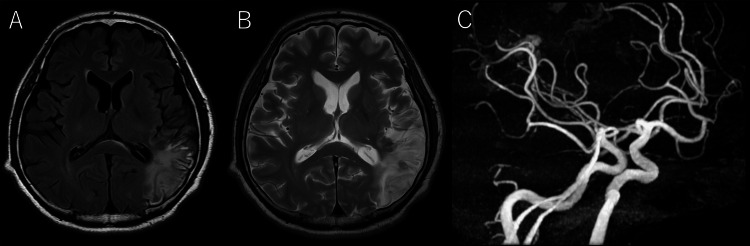
Post-coil embolization head MRI/MRA. (A) FLAIR image and (B) T2-weighted image reveal no new cerebral infarcts. (C) MRA confirms the complete obliteration of the aneurysm. MRA: Magnetic Resonance Angiography; FLAIR: Fluid-Attenuated Inversion Recovery image.

Postoperative MRI revealed no new cerebral infarctions (Figures 5A-5B), and MRA confirmed the absence of residual blood flow within the aneurysm (Figure 5C).

After completing a rehabilitation program, the patient was discharged on day 110 with a modified Rankin Scale score of 2. At an 18-month follow-up, no recurrence of the aneurysm was detected.

## Discussion

IIAs are relatively rare, accounting for 0.5-6.5% of all intracranial aneurysms [4, 12-14]. The precise mechanism underlying the spread of infection sources is uncertain but is thought to involve three possible pathways: endovascular, extravascular, and cryptogenic. The most common route is the endovascular pathway, where septic emboli originating from infected valve cusps in IE lead to IIAs of distal arteries [15-17]. The extravascular route involves direct spread from adjacent infected tissues, such as meningitis, cavernous sinus thrombophlebitis, orbital cellulitis, or post-neurosurgical infections, and typically affects proximal vessels passing through the infected area [15-17]. In the cryptogenic route, the mechanism is unknown and no systemic infection is evident [4, 6]. The MCA is the most frequently affected site, with 56.9% of cases. The morphology of IIAs is often fusiform, followed by saccular. Most IIAs are small to medium in size, with 44.2% measuring less than 5 mm, 30.8% between 5 and 10 mm, and 25.0% exceeding 10 mm, but their size can increase rapidly, up to several centimeters [4]. IE is the most common underlying cause of IIAs, accounting for approximately 70% of cases. The primary causative organisms are Viridans Streptococci and *Staphylococcus aureus* [4, 6, 14]. Although IIAs are reported in 2-10% of patients with IE, this prevalence may be underestimated due to spontaneous resolution or shrinkage following antibiotic treatment. Given this high prevalence and rapid, unpredictable progression, many literatures recommend routine screening for IIAs in IE patients, particularly within the first five weeks after symptom onset, as this is the period of highest risk for IIA formation and rupture [4, 18]. DSA is considered the most sensitive imaging modality for detecting IIAs, particularly small lesions, but its invasive nature limits its utility in critically ill patients. Although MRA and CTA cannot serve as perfect replacements, they are less invasive alternatives [4, 19].

In this case, the aneurysm was located in the artery responsible for the cerebral embolism and was not identified during the initial vascular screening. The patient might have undergone cardiac surgery while the aneurysm was likely forming. This underscores the importance of serial imaging evaluations in IE patients, as aneurysms may develop or enlarge after initial imaging, necessitating repeated assessments before any surgical intervention.

Currently, no randomized controlled trials or clear guidelines exist for the management of IIAs [4, 13].

Treatment options include antibiotic therapy, surgical intervention, and endovascular treatment (EVT). Alawieh AM et al.'s systematic review proposed treatment strategies based on factors such as rupture status, the necessity for cardiac surgery, patient surgical tolerance, the presence of hematomas causing mass effects, and the need for bypass surgery [4]. For unruptured IIAs, conservative therapy has traditionally been the treatment of choice [6]. However, mortality rates with antibiotic management alone remain significant, reported at 17.1% for unruptured IIAs and 25.8% for ruptured IIAs. In contrast, early surgical intervention is associated with substantially lower mortality rates, reported at 0% for unruptured IIAs and 6% for ruptured IIAs, highlighting the need for early surgical or endovascular intervention [13].

In recent years, EVT has gained prominence due to favorable outcomes, especially in patients with poor cardiac function or in cases involving distal aneurysms that are challenging to access surgically [18]. Endovascular procedures include coil embolization and parent artery occlusion (PAO) using coils or liquid embolic agents [6]. While PAO achieves higher aneurysm occlusion rates, it carries significant risks, including ischemic stroke and complications related to catheter removal or vessel injury [14]. Coil embolization is effective for obliterating the aneurysm sac while preserving the parent artery, but it comes with risks such as rupture during coil placement due to the distally located IIAs and the fragile vessel wall of the parent artery, coil compaction, and recanalization [8].

Concerns regarding the introduction of foreign objects into an infected site, potentially exacerbating infection or causing abscess formation, have not been proven. No documented cases of infectious complications or brain abscesses related to EVT in infective endocarditis (IE)-associated IIAs have been reported [20-22]. It has been suggested that septic emboli predominantly occur in the acute phase, whereas IIAs develop during the subacute phase when the patient is generally aseptic at the time of detection [23].

In this case, the patient required anticoagulation therapy following mitral valve repair and presented with a saccular aneurysm without mass effect. Considering these factors, coil embolization was chosen. To mitigate the risk of device-related infections, intravenous antibiotics were administered for two months preoperatively. The procedure successfully obliterated the aneurysm while preserving the parent artery, with no subsequent ischemic events or recurrence observed.

To our knowledge, including the present case, a total of 16 cases (involving 18 aneurysms) of unruptured IIAs treated with coil have been reported across 11 studies [15, 19, 22-30] (Table 2).

**Table 2:** Summary of coil embolization for unruptured infectious cerebral aneurysms. NA: Not available; IE: Infective endocarditis; MCA: Middle cerebral artery; ACA: Anterior cerebral artery; PCA: Posterior cerebral artery; EVT: Endovascular treatment; NBCA: N-butyl cyanoacrylate; BOT: Balloon occlusion test; GOS: Glasgow Outcome Scale; mRS: Modified Rankin Scale. *The age was not reported; the value of n=4 refers to four patients included in the analysis whose ages were unspecified.

Author (year)	Age	Sex	Presenting Symptoms	Imaging findings on admission	Etiology (Organism）	Aneurysm Location/Size	Aneurysm Type	Duration of Antibiotic Treatment Before EVT	EVT	Complete Obliteration	Complication/Outcome
Asai T, et al. (2002) [29]	21	M	Hemiparesis, dysarthria	CI with right MCA occlusion	IE (Streptococcus uberis)	Left distal MCA (M3)	NA	6 weeks	Trapping (Coil with NBCA)	Yes	No / Hemiplegia was improved
Chapot R, et al. (2002) [24]	55	M	NA	SAH with left distal MCA	IE (Streptococcus uberis)	Right proximal MCA	Saccular	10 weeks	Selective	Yes	No/ Normal
Nakahara I, et al. (2006) [25]	56	M	Low grade fever	Unruptured right PCA aneurysm	IE (Streptococcus oralis)	1: Right distal PCA/ 9.2 mm 2: left distal ACA/ 5.7 mm	NA NA	1: 3+ weeks 2: 6+ weeks	1: Selective 2: Trappping	Yes	No/ No neurologic deficit
Wajnberg E, et al. (2008) [26]	39	M	CN III palsy	NA	IE	BA Tip	NA	NA	Selective	Yes	No/ GOS 5
Martindale JL, et al. (2011) [27]	33	F	Fever, right arm numbness, confusion	CI with left MCA aneurysm	IE (Streptococcus sanguinis)	Left distal MCA (M3)/ 4 mm	Fusiform	3+ days	Selective	Yes	No/ Mild right hand paresthesias
Lv N, et al. (2016) [28]	45	M	Headache	NA	IE	1: Left distal MCA (M3)/ 5.0 mm 2: Left distal MCA (M4)/ 7.8 mm	NA	NA	NA	Yes	No/ Good
Matsubara N, et al. (2015) [23]	27	F	Mass effect	NA	Sepsis (Enterococcus)	ICA cavernous/ 15 mm	Fusiform	NA	Trapping	Yes	No/ ｍRS 1
Matsubara N, et al. (2015) [23]	51	F	Mass effect	NA	Meningitis (Streptococcus)	ICA cavernous 5.5 mm	Saccular	NA	Selective	Yes	No/ ｍRS 0
Esenkaya A, et al. (2016) [15]	40	M	NA	CI in deep parietal lobe	IE	Left MCA (M2-M3)/ 8 mm	Saccular	NA	Selective	Yes	No/ ｍRS 0
Nonaka S, et al. (2016) [19]	47	M	Hemianopia	CI with left PCA aneurysm	IE (Methicillin-resistant Staphylococcus aureus)	Right distal PCA (P3-4)/ 10×8 mm	Saccular	NA	Trapping after BOT	Yes	No/ ｍRS 0
Ando K, et al. (2019) [30]	43	M	Sudden headache, sensory aphasia	Partially thrombosed aneurysm	IE (Enterococcus faecalis)	Left proximal MCA/ 50ｍｍ	Fusiform	NA	Trapping with revascularization	Yes	No/ ｍRS 3
Lai PM, et al. (2022) [22]	n= 4※	NA	NA	NA	IE	NA	NA	NA	Selective	Yes	NA/ NA
Present case	54	M	Fever, impaired consciousness	CI	IE (Methicillin-sensitive Staphylococcus aureus)	Left MCA (M2-M3)/ 5.4× 3.8 mm	Saccular	8 weeks	Selective	Yes	No/ mRS2

The mean age at diagnosis was 43 years (median 44; range 21-56 years), with a male-to-female ratio of 3:1 (data unavailable for four cases). The most common etiology was IE (14 cases), with sepsis and meningitis accounting for one case each. The middle cerebral artery (MCA) was the most frequently affected location (6 cases), followed by the posterior cerebral artery (2 cases), basilar artery (1 case), and cavernous internal carotid artery (2 cases). The mean aneurysm size was 11.4 mm (median 7.8 mm; range 4-50 mm; data unavailable for seven cases). Saccular morphology was observed in 5 cases, fusiform in 3 cases, and unspecified in 10 cases. The interval between antibiotic initiation and EVT ranged from 3 days to 10 weeks (data unavailable for 11 cases); in one case it was three days, but in the rest it was three weeks or more. Coil embolization was performed in 11 aneurysms, and PAO in five cases. All cases demonstrated favorable outcomes without complications. Notably, small aneurysms (<10 mm) were more likely to be treated with coil embolization.

Based on the existing literature, coil embolization is a viable management option for unruptured IIAs smaller than 10 mm, provided the patient has undergone at least three weeks of adequate antibiotic therapy. This approach minimizes the risk of procedural complications while effectively managing the aneurysm, offering a balance between efficacy and safety.

This study has several limitations. First, the small sample size limits comprehensive statistical analyses. Second, incomplete information in reported cases restricts detailed evaluation of factors influencing treatment outcomes and complications. Third, reporting bias inherent to literature reviews cannot be excluded. To address these limitations, future prospective studies or multicenter collaborative research with larger sample sizes are required to establish optimal treatment strategies for unruptured IIAs.

## Conclusions

In this case report, EVT with coil embolization was successfully performed for an IIA associated with IE, while preserving the parent artery, resulting in a favorable clinical outcome. Several factors likely contributed to this success, including the small size of the aneurysm (<10 mm), its saccular morphology, and its accessible location. Additionally, the administration of appropriate antibiotic therapy both before and after the procedure was critical in minimizing infection-related complications. This case highlights the importance of a tailored treatment strategy for individual patients.
